# Plant-bacterium interactions analyzed by proteomics

**DOI:** 10.3389/fpls.2013.00021

**Published:** 2013-02-15

**Authors:** Amber Afroz, Muzna Zahur, Nadia Zeeshan, Setsuko Komatsu

**Affiliations:** ^1^Department of Biochemistry and Molecular Biology, Nawaz Sharif Medical College, University of Gujrat, Hafiz Hayat Campus GujratGujrat, Pakistan; ^2^National Institute of Crop Science, National Agriculture and Food Research OrganizationTsukuba, Japan

**Keywords:** pathogen associated molecular patter, pattern recognition receptors, effector triggered immunity, virulence, pathogenic bacteria, symbiotic bacteria, proteomics

## Abstract

The evolution of the plant immune response has resulted in a highly effective defense system that is able to resist potential attack by microbial pathogens. The primary immune response is referred to as pathogen associated molecular pattern (PAMP) triggered immunity and has evolved to recognize common features of microbial pathogens. In response to the delivery of pathogen effector proteins, plants acquired R proteins to fight against pathogen attack. R-dependent defense response is important in understanding the biochemical and cellular mechanisms and underlying these interactions will enable molecular and transgenic approaches for crops with increased biotic resistance. Proteomic analyses are particularly useful for understanding the mechanisms of host plant against the pathogen attack. Recent advances in the field of proteome analyses have initiated a new research area, i.e., the analysis of more complex microbial communities and their interaction with plant. Such areas hold great potential to elucidate, not only the interactions between bacteria and their host plants, but also of bacteria-bacteria interactions between different bacterial taxa, symbiotic, pathogenic bacteria, and commensal bacteria. During biotic stress, plant hormonal signaling pathways prioritizes defense over other cellular functions. Some plant pathogens take advantage of hormone dependent regulatory system by mimicking hormones that interfere with host immune responses to promote virulence (vir). In this review, it is discussed the cross talk that plays important role in response to pathogens attack with different infection strategies using proteomic approaches.

## Introduction

Unlike plant and animal cells, most bacteria are exposed to a constantly changing physical and chemical environment. Phylogenetic diversity of plant-associated bacteria (PAB) can categorize them in to commensals (acquire nutrients from the plant without damaging), mutualists (positively influence plant health), and pathogens (damage plant) (Newton et al., 2010). Notably pathogenic, commensals, or mutualists bacteria have developed strategies to interact with plants overlap, exceptionally modified physiology that accounts for individual need (Martin et al., 2003; Boller and Felix, 2009). Bacteria react to changes in their environment through changes in patterns of structural proteins, transport proteins, toxins, and enzymes, which adapt them to a particular habitat (Boller and Felix, 2009). Enzymes are either constitutive in nature (always produced by cells independently of the composition of the medium) or inducible (produced in cells in response to the end product of a pathway). Regulation of enzyme activity which is mainly operates to regulate biosynthetic pathways and catabolites repression is considered a form of positive control because it affects an increase in rates of transcription of proteins (Deutscher, 2008).

Plant immunity that recognize pathogens by membrane proteins is termed as pattern recognition receptors (PRRs), which recognize pathogen associated molecular pattern (PAMP) and is basis of plant innate immunity (Gomez-Gomez and Boller, 2000). PAMP recognition also results in plant systemic acquired resistance and production of resistance (R) proteins leading to effector triggered immunity (ETI), which is often accompanied by the hypersensitive response (HR), and programmed cell death (Jones and Dangl, 2006). Over the past 10 years, many R genes have been isolated that confer resistance to various pathogens including virus, bacteria, fungi, or nematodes (Martin et al., 2003). Based on predicted protein sequences, these R gene products are divided into intracellular protein kinases (Pto), proteins with an extracellular leucine-rich repeat (LRR) domain and a cytoplasmic protein kinase region (e.g., *Xa21*), intracellular proteins containing a region of a LRRs and a nucleotide binding site (RPS2, RPM1), intracellular proteins containing a region of homology to the Toll/IL-1R proteins in addition to LRRs and a nucleotide binding site (e.g., N, L6, RPP5), and proteins with LRRs that appear to encode membrane-bound extracellular proteins (e.g., Cf-4, Cf-9) (Chisholm et al., 2006; Zhang et al., 2012).

Proteomic analyses have made possible the analysis of complex microbial communities, which had great potential to elucidate not only the interactions between bacteria and their host plants, but also of bacteria-bacteria interactions. Proteomic reference data sets were established for various PAB, via two-dimensional polyacrylamide gel electrophoresis (2-DE) gels, resulting in a few hundred identified proteins (Rosen et al., 2004; Chung et al., 2007), or multidimensional liquid chromatography-tandom mass spectrometry (LC-MS/MS) techniques resulting in the detection of more than 1000 proteins (Anderson et al., 2006; Bosch et al., 2008). Era is followed by gel free proteomics but quantitation procedures have to be optimized before the gel-based proteomics can be replaced by gel free procedures (Washburn et al., 2001).

Complete genome sequence of a *Xylella fastidiosa* is available which can be very helpful in genomics and proteomic studies of plant-bacterium interactions (Simpson et al., 2000; Bagnarol et al., 2007). More genomic data is needed for pathogenic and symbiotic bacteria to understand the molecular signaling pathways involved in plant-bacterium interactions. *P. syringae* and *Xanthomonas campestris* are important PAB on the grounds of agricultural importance and intensity of scientific research. Both are pathogenic on the model plant *Arabidopsis* (Jones et al., 2006a; Andrade et al., 2008). Pathogenic and mutualist PAB had been extensively studied (Rosen et al., 2004; Jacobs et al., 2012). The process of mutualism involved the significant change in the metabolism of both the mutualists and host, which involves a change in plant cell metabolism to support ATP synthesis and nitrogen fixation by the mutualist for nodule development (Delmotte et al., 2010). Transcriptomics data shows that pathogenic bacteria involve the hypersensitive reaction and pathogenicity (hrp) gene and different secretion systems (SS) for colonization and damaging host cells (Buttner and Bonas, 2010). In these review proteins of PAB bacteria have been compared. They typically exchange signals with their hosts and possess a range of specific adaptations for plant colonization. The importance of proteomics is explored to understand the molecular mechanism, by which bacteria adapt to live in association with plants for evolution of symbiosis and pathogenesis. This will opens up new research areas concerning protein-based plant-microbe communication and provides important information regarding the manipulation of gene expression of specific proteins with the purpose of modifying plant behavior related to compatible or incompatible interactions.

## PAMP recognition by pattern recognition receptors

PAMPs constitute the first layer of plant innate immunity, and lacking of its recognition can lead to enhanced disease susceptibility. PAMPs are ideal elicitors for “non-self” surveillance systems such as chitin, ergosterol, and transglutaminase from fungi, and/or lipopolysaccharides and flagellin from bacteria, stimulate plant encoded PAMP receptors (Chisholm et al., 2006). Intracellular responses associated with PAMP-triggered immunity (PTI) including rapid ion fluxes across the plasma membrane, mitogen-activated protein (MAP) kinase activation, production of reactive oxygen species (ROS), and rapid changes in gene expression and cell wall reinforcement. Suppression of PTI may be achieved by secretion of virulence (vir) effectors by the pathogens or by suppression of plant signaling. ETI is accompanied by production of R protein or HR, illustrating the dynamic co evolution between plants and pathogens (Jones and Dangl, 2006).

Flagellin, elongation factor (EF) Tu, peptidoglycan, lipopolysaccharide, and bacterial cold shock proteins are important PAMPS and plant responses induced by them are referred to as “basal” defenses (Newton et al., 2010). Upon recognition of highly conserved amino terminus of flagellin (flg22), flagellin sensing 2 (FLS2) induces a suite of defense responses, including MAP kinase signaling, transcriptional activation, and deposition of callose, a putative physical barrier at the site of infection (Gomez-Gomez et al., 1999). EF Tu potent bacterial PAMP in *Arabidopsis* and other members of the Brassicaceae family, serves as an adhesion factor at the bacterial surface, in addition to its primary role in translation (Boller and Felix, 2009). Asparate oxidase is required for PAMP-triggered RBOHD-dependent (responsible for stomatal closure) ROS burst and stomatal immunity against the *P. syringae* (Macho et al., 2012). The LRR receptor kinases, EF-Tu receptor and FLS2 are PRRs, contributing to disease resistance against the hemibiotrophic bacterium *P. syringae* (Roux et al., 2011).

The plant hormones, salicylic acid (SA), jasmonic acid (JA) and ethylene, have emerged as key players in the signaling networks involved in plant immunity. Rhamnolipids are glycolipids produced by bacteria and are involved in surface motility and biofilm development and are considered as PAMPS. Ethylene is found to be involved in rhamnolipid-induced resistance to *H. arabidopsidis* and to *P. syringae* whereas JA is essential for the resistance to *B. cinerea*. SA participates in restriction of all bacterial and fungal pathogens, so involving in broadly rhamnolipid mediated immunity (Sanchez et al., 2012). PAMPS are sometimes succeeded and sometimes fails to induce PTI depending upon the type of compatible and non-compatible interactions. Flagellin is capable of suppressing HR via PTI induction during an incompatible interaction (Wei et al., 2012).

Type III secretion system (T3SSs) were essential components of two complex bacterial machineries: the flagellum, which drives cell motility and the non-flagellar T3SS (NF-T3SS), which delivers effectors into eukaryotic cells (Mudgett, 2005). *P. syringae* use T3SS to deliver up to 40 effector proteins into host cells, inhibiting basal host defense responses, such as HR (McCann and Guttman, 2008).

PAMP induced PTI serves as a primary plant defense response against microbial pathogens, with MAP kinase cascade downstream of PAMP receptors. LRR-RLKs including PSKR1 act as PTI against pathogenic bacteria, and plants expressing this gene show enhanced PAMP responses and less lesion formation after infection with the bacterial pathogen *P. syringae* via jasmonate signaling pathway (Mosher et al., 2013). Peptidoglycan, an important PAMP from *Staphylococcus aureus* results in PTI, such as medium alkalinization, elevation of cytoplasmic calcium concentrations, nitric oxide, and camalexin production, and the post-translational induction of MAP kinase activities (Gust et al., 2007).

PAMP recognition also results in plant systemic acquired resistance and production of R proteins such as SUMM2 that becomes active when the MAP kinase cascade is disrupted by pathogens, leading to ETI (Zhang et al., 2012). In rice, the LRR-RK Xa21 confers resistance to *Xanthomonas oryzae* pv. oryzae strains carrying the Avr gene AvrXa21 (Song et al., 1995). AvrXa21 as a type I secreted sulfated peptide, is conserved among all *Xanthomonas* strains sequenced (P. Ronald, pers. communication), suggesting that *AvrXa21/Xa21* constitutes a PAMP/PRR perception system (Lee et al., 2008). Although many PAMPs recognized by plants have been described, number of known PRR and PTI is still in its infancy, constituting a highly active and competitive field of research.

## Proteome analyses of plant associated bacteria

PAB either they are pathogenic or symbiotic bacteria adhere to plant surfaces, invade the intercellular space of the host tissue, counteract plant defense systems and acquire nutrients. However either there is establishment of a pathogenic interaction or mutualist relationship develops. Cell surface proteins such as adhesions, polysaccharides, lipopolysaccharides, and degradative enzymes enable the degradation of the plant cell wall and also result in basal plant defenses (Newton et al., 2010). Proteins of PAB are studied either in planta, by means of bacterial responses to selected biomolecule or plant extracts, synthetic media, or secretome analysis to study the vir factor of the bacterial pathogens (Guerreiro et al., 1997; Corbett et al., 2005; Gourion et al., 2006; Chung et al., 2007). All studies had helped to study plant-microbe interactions.

*X. fastidiosa*, whose genome was fully sequenced leads to a clearer understanding of the biology of phytopathogenic organism at both the genomic and proteome levels (Simpson et al., 2000). The cellular and secreted protein profiling of pathogenic bacteria *X. fastidiosa*, led to the identification of proteins involved in cellular adhesion systems, proteases, antioxidant, and toxins (Smolka et al., 2003). These proteins can be the candidates for understanding of molecular mechanism of disease cycle of citrus variegated chlorosis and Pierce's disease in grapevine caused by *X. fastidiosa*. In non-virulent strain of *E. chrysanthemi* (*opg* mutant), differential expression of protein is not restricted to the envelope, but affects general metabolism such as membrane lipid composition, protein folding, carbohydrate catabolism, and protein degradation (Bouchart et al., 2007). The secretome of *X. campestris* pv. *campestris* using 2-DE and matrix-assisted laser desorption/ionization time of flight (MALDI-TOF) MS resulted in the identification of 87 proteins known to be involved in degradative activities and important for the infection of susceptible plant hosts (Watt et al., 2005).

Diverse pathogenic responses are reported due to differences in induction of vir between closely related strains. Chung et al. (2007) reported that degradative enzymes and virulent secreted proteins were only seen in the pathogenic *X. campestris*. Moreover, HtrA was identified in the virulent *X. campestris* pv. campestris strain (Chung et al., 2007). Unique proteins involved in T3SSs activities and iron uptake were only consistently expressed in the virulent strain of *P. stewartii* (Bouchart et al., 2007; Wu et al., 2007). Proteins accumulated in T3SS cascade were related to iron uptake, motility, adhesion, metabolism, and transcriptional regulation (Wu et al., 2007). The siderophore-based iron uptake system is a common mechanism employed by gram-negative pathogenic bacteria.

Bacteria are known to react to a number of signaling molecules released by plants by specific gene expression. In *Rhizobium leguminosarum*, the proteins of cells were analyzed in the presence and absence of 7, 40-dihydroxyflavone. Proteins related to amino acid metabolism and transport, flagellin, energy, translation and structure were induced (Guerreiro et al., 1997; Table 1). In *Agrobacterium tumefaciens* acetosyringone is sensed by the virA/G two component systems and induces expression of the vir genes encoded on the Ti plasmid (McCullen and Binns, 2006). Vir proteins were reported to have T4SSs for the delivery of bacterial T-DNA into the host cell and to have molecular chaperone functions as an assembly factor (McCann and Guttman, 2008; Lai et al., 2006). Incubation of *A. tumefaciens* in the presence of cut root material led to the induction of ribosomal protein, stress related chaperones, ABC transporters and sugar binding proteins indicating the significance of protein modifications in the interactions of Agrobacteria with plants (Rosen et al., 2003). Addition of suberin to *Streptomyces scabies* induced 17 proteins linked to primary, secondary metabolism and stress related pathway (Lauzier et al., 2008). Expression of superoxide dismutase (SOD) was enhanced in *Frankia* strains and *Streptomyces coelicolor* with plant extracts (Langlois et al., 2003; Bagnarol et al., 2007). Addition of carbohydrates such as rhamnose and ferulic acid to medium induce plant phenolic compound (rhamno galacturonate lyase and esterase) in *D. dadantii* (Kazemi-Pour et al., 2004). Other identified proteins of *E. chrysanthemi* were cellulase, flagellin, pectinases, endopectate lyases, pectin acetylesterases, pectin methylesterase, and polygalacturonase (Kazemi-Pour et al., 2004). Proteins involved in signal transduction, translation, ribosomal proteins and biogenesis, inorganic ion, lipid, amino acid, energy, transport, and metabolism were induced by plant extracts (Langlois et al., 2003). These results suggest that root exudates provide additional carbon sources to the bacteria and that physiological adaptation are required for efficient bacterial growth in the presence of plants. Differential proteins involved in carbohydrates, lipids, purines, metabolism, transcription, coenzymes, chaperones and iron transport regulation, essential for nitrogen fixation, seem to be strain dependent (Dixon and Kahn, 2004).

**Table 1:** **Expression profiling of bacterial strains in differential medias/plant associated bacteria as pathogens, symbiotic or epiphytes**.

**Organism**	**Pathogen**	**Proteomic techniques**	**No. of IP**^**1**^	**Virulence factor, secretion systems, proteins expressed *in vivo* or under defined medias in different bacteria**	**References**
*Xanthomonas campestris* pv. Campestris/parasite	*Brassica oleracea*, (cv. Coracao de boi) 4–6 days after inoculation (DAI)	2-DE, MALDI-TOF/TOF	21	Proteins from young leaves of susceptible *Brassica* cv infiltrated with *X. campestris*: aspartate semialdehyde dehydrogenase, elongation factor thermo unstable, phosphomannose isomerase, adenine triphosphate (ATP) synthase, ribosomal protein, chaperonin, phosphoglycerate kinase, and ATPase.	Andrade et al., 2008
*Frankia* sp. Strains M16467/symbiont	*Morella cerifera and Myrica gale*	2-DE, MALDI-TOF MS	50	With and without *M. cerifera, M. gale* seed phenolic extracts. Twenty proteins with increased abundance: signal transduction mechanisms, translation, ribosomal structure and biogenesis, chaperone heat shock protein, translation, amino acid and lipid transport, and metabolism. Thirty proteins with decreased abundance: Post-translational modification, chaperones, replication, transcription, translation, recombination, repair, energy production, conversion, lipid, inorganic ion, coenzyme, nucleotide, carbohydrate and secondary metabolite (transport and metabolism), cell wall biogenesis, and defense.	Bagnarol et al., 2007
*Pseudomonas savastanoi* pv. savastanoi/6 weeks after infection	*Olea europaea* subsp. europaea cvs Galega and Cordovil de Serpa	2-DE, MALDI-TOF MS	7	Proteins accumulated *in vivo* in *O. europaea* stems after infection by *P. savastanoi*: aconitate hydratase, tellurium resistance protein, enolase, hypothetical protein, and calcium dependent protein kinase.	Campos et al., 2009
*X. campestris* pv. *campestris* strain	*X. campestris* pv. *campestris* strain 11A/17	2-DE, MALDI-TOD MS	281	General protein accumulation in *X. campestris*: energy, metabolism, carbohydrate, lipid, protein and cofactors and vitamins metabolism, biosynthesis of secondary metabolites, transcription, translation, replication and repair, membrane transport, signal transduction, cell motility (22 higly abundant proteins of virulent vs. avirulent: aspartate semialdehyde dehydrogenase, cold shock protein, elongation factor, cellulase, L-isoaspartate protein carboxyl methyltransferase, malate dehydrogenase, 50S ribosomal protein, fumaryl acetoacetate hydrolase, peptidyl-prolyl *cis-trans* isomerase, 10 kDa chaperonin).	Chung et al., 2007
*Bradyrhizobium Japonicum/Symbiont*	*Glycine max*/21 DAI	LCMS/MS, LTQ-Orbitrap MS	3587 genes/proteins	Proteins involved in translation, post transcriptional regulation, nitrogenase complex, aspartate amino transferase, carbon metabolism, translation, and nucleic acid metabolism.	Delmotte et al., 2010
*S. scabies*	Wild type/*tatC mutant* strains	2-DE, MALDI-TOF TOF MS	73	Tat dep virulence (vir) factors: glycosyl hydrolase domain, putative alpha-L-fucosidase, ABC-type Fe^3+^ transport system, periplasmic component, glycosyl hydrolase domain, hydrolase of the α/β superfamily, lipoprotein, spermidine/putrescine transporter peptide- binding protein, and rhamnosidase.	Joshi et al., 2010
*Sinorhizobium meliloti/Symbiont*	*Medicago truncatula* and *Melilotus alba*	2-DE, MALDI-TOF MS	420/700	Proteomes of the nodule bacteria compared to *S. meliloti in vitro*: transport proteins (ABC transporters, leucine and Iron binding protein), vitamin synthesis, stress-related processes, superoxide dismutase, betaine aldehyde dehydrogenase, heat shock protein, energy, GTP-binding proteins, oxidoreductase NAD protein, catalase, cell division, ribosomal protein, deoxyribonucleic acid transcription, translation, and central metabolism.	Djordjevic, 2004
*Methylobacterium extorquens/epiphyte*	*A. thaliana*	2-DE, LC MS/MS	45	PhytR regulated proteins: alcohol dehydrogenase, catalase, reactive oxygen species, stress proteins, lactoylglutathione lyase, dioxygenase, glutathione-dependent formaldehyde dehydrogenase, malyl lyase, malate dehydrogenase, glutathione-dependent formaldehyde dehydrogenase, haloacetate dehalogenase, transcription elongation factor, lipid metabolism, propionyl-CoA carboxylase, and dehydrogenases reductases.	Gourion et al., 2006
*Rhizobium*. *R. leguminosarum* bv. *trifolii* strain ANU843/symbiont	flavonoid 7,4 dihydroxyflavone (released due to legume nodulation)	2-DE, Protein sequencer	12	*R. leguminosarum* grown in presence and absence of 7,4-dihydroxyflavone: amino acid metabolism and transport, flagellin, protein translation and structure, energy, DnaK, NodE, and NodB.	Guerreiro et al., 1997
*Azoarcus* sp. strain BH72/endophyte	PilR mutant (necessary for endophytic growth)	2-DE, MALDI-TOF MS/MS, LC-MS/MS	785/30 abundant	PilR mutant proteins: amino acid and energy metabolism, chaperones, iron metabolism and storage, ATP synthase, ABC transporter, heat shock protein, pyridoxal phosphate, DNA, RNA polymerase, *S*-adenosyl-L-homocysteine hydrolase, fumarate hydratase, and ATP-dependent protease.	Hauberg et al., 2010
*Burkholderia glumae* (grain and seedling rot in rice, bacterial wilt in many crops)	*Under hrpB expression (induced after attack to plant)*	2-DE, ESI-MS/MS	34 secretory/12 cytoplasmic	Induction of type III secretion system (T3SS), acetylglutamate kinase, ribosomal protein, transcription elongation factor, Acetyl-CoA biotin carboxyl carrier protein, chaperone protein DnaK, lipoprotein, ATP synthase, phenylalanyl-tRNA synthase, and alkyl hydroperoxide reductase.	Kang et al., 2008
*Rhizobium leguminosarum* Biovar viciae (symbiont)	*Pisum sativum* and *Vicia cracca*	Microarray		Genes expressed related to tricarboxylic acid cycle, Succinate, pyruvate/inositol catabolism, α aminobutyrate metabolism, regulators, exported and cell surface molecules, multi drug exporters, and heat and cold shock proteins (early induced) *fix* genes, *nif* (late induced).	Karunakaran et al., 2009
*Erwinia chrysanthemi (soft rot)*	*Chrysanthemum* leaves extract	2-DE, MALDI-TOF MS	25	Endopectate lyases, pectin acetyl esterases, pectin methylesterase, poly galacturonase, flagellin, and elongation factor.	Kazemi-Pour et al., 2004
*Agrobacterium tumefaciens*	200 μM acetosyringone (As)	2-DE, MALDI-Q-TOF MS	11	As induced vir proteins, type IV secretion system (T4SS), newly As-induced proteins regulated by the virA/virG, an unknown protein Y4mC, and heat shock protein.	Lai et al., 2006
*Streptomyces coelicolor*	*Lemna minor fronds*	2-DE, MALDI-TOF MS	31	Differential proteins of *S. coelicolor* with and without *L. minor* fronds in minimal medium: proteins related to energy, metabolism, protein synthesis, proteins involved in the acquisition of carbon, stress-induced (chaperonin, ATP-GTP binding protein, tellurite resistance protein, and Fe superoxide dismutase).	Langlois et al., 2003
*S. meliloti* strain 2011	*Medicago truncatula*/3–6 D for drought	LC MS/MS	377	Proteins accumulated in *M. truncatula in vivo* with *S. meliloti* under drought: protein synthesis/degradation, stress proteins, RNA regulation, secondary metabolism, signaling, amino acid carbohydrate, nucleic acid/hormone metabolism, glycolysis/tricarboxylic acid cycle.	Larrainzar et al., 2007
*Streptomyces scabies*	potato suberin, lipidic plant polymer	2-DE, Protein sequencer MS/MS	19	Differential proteins in *S. scabies* in minimal media with and without 0.2% potato suberin: proteins with increased abundance in response to suberin (Nucleotide and amino acid metabolism, glycolysis/tricarboxylic acid cycle enzymes, chaperonin, phenylacetaldehyde dehydrogenase, fructose-bisphosphate aldolase, triose phosphate isomerase, ABC transporter, ATPase subunit, lipoprotein, and serine hydroxymethyl transferase).	Lauzier et al., 2008
*Frankia* sp. strain CcI3 (Symbiont)	*Casuarina cunninghamiana and C. glauca/Alnus incana* and *Elaeagnus angustifolia*	2-DE, MS/MS, LC MS/MS	73/53	*Frankia strains* in root nodules of *Alnus incana* and *Elaeagnus angustifolia* (cell wall/growth enzymes, solute-binding proteins (amino acids, peptides, inorganic ions) (ABC transporter, molybdate binding proteins, Leu/Ile/Val/Glu/Thr-binding protein precursor, hydrolytic enzymes, proteins involved in cell processes (DNA polymerase, signal transduction histidine, and T2SS protein).	Mastronunzio et al., 2009
*Pectobacterium atrosepticum* (Pathogen in potato)	Potato stem/tubers extract	2-DE, MALDI-TOF MS	40	Abundant proteins in minimal medium with potato tuber extracts: (T3SS protein, pectin enzymes, vir protein Svx, flagellar hook associated protein, endo-polygalacturonase, dihydrolipoamide dehydrogenase, hexosaminidase, pectate lyase, fructose bisphosphate aldolase, ABC transporter, glyceraldehyde 3-phosphate dehydrogenase, and chaperones).	Mattinen et al., 2007
*Ralstonia solanacearum* UW551 (phylotype II) and GMI1000	*S. lycopersicum* cv. Bonny Best	Microarray	109 by HrpB	Enhancement of hrpB regulated genes via T3SS, sucrose uptake and catabolism via SCR ABC, sucrose dependent phosphoenol pyruvate-carbohydrate phosphotransferase, glycolysis enzymes, cell wall-degrading enzymes, exopolysaccharide, reactive oxygen species, inorganic, organic and aminoacid and nucleic acid metabolic enzymes, tricarboxylic acid cycle intermediates, and pentose phosphate pathway.	Jacobs et al., 2012
*B. japonicum* USDA110 (Symbiont)	*G. max* L. Merrill cultivar Akishirome. 7–49 DAI	2-DE, MALDI-TOF MS	275	Proteins accumulation related to transcription, Nif and Fix proteins, translation, protein folding, and degradation, synthetic enzyme of the poly-beta-hydroxybutyrate, solute transporter, and elongation factor-thermo unstable.	Nomura et al., 2010
*A. tumefaciens*. C58 strain ATCC 33970	*Solanum lycopersicum* cv. Rutgers stem, roots	2-DE, MALDI-TOF MS	30	Augmentation of ABC transporter, ATPase protein, alcohol dehydrogenase, enoyl CoA hydratase/isomerase, aldehyde dehydrogenase, protein-L-isoaspartate O-methyltransferase, acetyl COA carboxylase, ribosomal proteins (chaperonin, hydrolases, and sugar-binding protein), and aldolase.	Rosen et al., 2003
*B. japonicum* USDA110	HM medium (Cole and Elkan, 1973)/root nodule residing *B. japonicum*	2-DE, MALDI-TOF MS	1200	Suppression of fatty acid, nucleic acid and cell surface synthesis, DNA metabolism-related proteins and proteolytic enzymes. Enhancement of translation, transcription related proteins, elongation factor, chaperones (heat shock protein, chaperonin), ATP synthase, DNA polymerase, and nitrogen metabolism proteins.	Sarma and Emerich, 2006
*B. japonicum* USDA110	*G. max*. cv. Williams 82. Nodule protein	2-DE, MALDI-TOF MS	180	Augmentation of proteins related to nitrogen, carbon metabolism, protein synthesis, scaffolding and degradation, cellular detoxification function (ATP synthetase, elongation factor ribosomal protein, chaperonin, heat shock protein), stress regulation, signaling communication. Decline of fatty acid and nucleic acid metabolism, solute transport (ABC transporter) proteins, protein synthesis, scaffolding and degradation, cellular detoxification, stress regulation and signaling communication.	Sarma and Emerich, 2005
*B. japonicum*	*G. max, Vigna unguiculata* and *Macroptilium atropurpureum*	LC-MS/MS	2000	Accumulation of housekeeping proteins, ribosomal proteins, ABC-type transporter sulfonate-binding protein, enoyl-CoA hydratase, transketolase, hydroxyphenylpyruvate dioxygenase, nitropropane dioxygenase.	Koch et al., 2010
*Xylella fastidiosa* strain 9a5c	Brazilian sweet orange	2-DE, MALDI-TOF MS	30	Synthesis of aconitate hydratase, DNAk protein, dihydrolipoamide dehydrogenase, lipase/esterase, 30S, 50S ribosomal protein, inosine-5′-monophosphate dehydrogenase, heat shock protein, peptidyl-prolyl cis-trans isomerase, ATP synthase, aspartate-B-semialdehyde dehydrogenase, alcohol dehydrogenase, fructose-bisphosphate aldolase, malate dehydrogenase, ABC transporter ATP-binding protein, chaperone, elongation factor, and RNA polymerase.	Smolka et al., 2003
*Xanthomonas citri* subsp. citri	TSE medium (with sucrose and glutamic acid) induce pathogenesity	2-DE, LC MS/MS	1702	Expression of Hrp gene dependent T3SS enzymes, tricarboxylic acid cycle, glycolysis/gluconeogenesis, pentose phosphate pathway, other sugar metabolism, urea cycle, pyrimidine and purine biosynthesis, fatty acid synthesis and degradation pathways, polyamine biosynthesis, DNA, RNA, protein metabolism, initiation, elongation, transcription, translation factors, cell structure and function, division, transport, pathogenesis and vir.	Soares et al., 2010
*X. campestris* pv. *campestris* B100		2-DE, MALDI-TOF MS	87	Accumulation of outer membrane proteins with signal peptide, ATP synthase beta chain, 30s ribosomal protein, metabolic proteins, protein maintenance and folding (chaperonin, DNA k), and degradive enzymes (cellulase, lipase).	Watt et al., 2005
*Pantoea stewartii* subsp. stewartii (Pnss) vir/P. stewartii subsp. indologenes (Pnsi) avir	Stewart's bacterial wilt and leaf blight of maize and sweet corn	2-DE, LC MS/MS, MALDI-TOF MS	21	Proteins accumulation in virulent verses avirulent strain: siderophore-based iron uptake system, motility, adhesion, and biofilm formation (flagellin B homolog), pilus adhesion, transdolase, secretory protein (T3SS protein, HrcJ, 60 kDa chaperonin), mannosyl transferase, transdolase, LysR, transcriptional activator belonging to the AraC family, ABC transporter.	Wu et al., 2007
*A. tumefaciens*		LC MS/MS	12	Enhancement of T4SS secretome proteins, virB, hemolysin- coregulated protein, periplasmic binding protein, amino acid-binding periplasmic protein, hemin-binding lipoprotein, and dipeptide protein.	Wu et al., 2008
*S. meliloti mutants* 2011-3.4 and 1021Δ*hfq*	Alfalfa	2-DE, MALDI-TOF MS	33	Proteins lower in abundance were sugar transporters, enzymes of central carbon metabolism, glycine betaine, electron transport chain, iron, sugar catabolism, biosynthesis of aminoacids, vitamins, purines and pyrimidines, chaperonin, heat shock protein, and ABC transporter. Proteins augmented were metabolism of nitrogen sources (mainly amino acids), glycine cleavage system, metabolic enzymes such as ornithine cyclodeaminase, arginase, adenosylhomocysteinase, and phosphoenol pyruvate carboxykinase.	Torres-Quesada et al., 2010

1 IP: Number of identified protein.

Abbreviations: DAI, days after inoculation; ATP, adenine triphosphate; As, Acetosyringone; vir, virulence; T3SS, Type III secretion system; T4SS, Type IV secretion system; 2-DE, two-dimensional polyacrylamide gel electrophoresis; MS, Mass spectrometry; MS/MS, Tandom Mass spectrometry; MALDI-TOF, Matrix-assisted laser desorption/ionization time of flight; LC, liquid chromatography.

Proteome analysis is very tricky when dealing with separation of bacteria from infected plants and additional steps are needed to avoid the changes in proteome map. Protocols had been proposed for the bacterial separation by density gradient centrifugation using percoll or sucrose gradients (Gourion et al., 2006; Nomura et al., 2010). Jacobs et al. (2012) discussed the transcriptomics profile of *R. solanacearum in vitro* and he discussed the importance of T3SS in vir cascade of *Ralsotonia* (45% regulated by HR and Hrp gene). Proteome analysis of pathogenic bacteria *X. campestris* pv. campestris in association with *B. oleracea* and *Pseudomonas savastanoi* pv. savastanoi resulted in comprehensive expression analysis including stress and metabolic proteins (Andrade et al., 2008; Campos et al., 2009). Increased levels of proteins involved in xanthan biosynthesis, stress response, and the metabolism were induced in *X. campestris* in planta conditions compared with *in vitro* grown cells (Andrade et al., 2008). Chaperonin is reported to be involved in stress responses and EF, which acts as an important PTI in the plants, is the key component of the translational machinery of bacteria. Xanthan is an extracellular polysaccharide probably responsible for disease symptom in planta growth via mucoid appearance of the bacterial colonies and wilting of host plants by blocking the water flow in xylem vessels (Buttner and Bonas, 2010).

The differential proteome analysis of in planta and *in vitro* grown cells of *Methylobacterium extorquens* resulted in the identification of 45 metabolic proteins and proteins involved in stress response such as the extracellular protease, SOD, catalases, and the DNA protection protein (Gourion et al., 2006). The protein analysis of cyanobacteria living in symbiosis revealed several adaptations to a symbiotic lifestyle, including an increase in proteins involved in energy production and nitrogen fixation. On the other hand, proteins involved in photosynthesis were decreased, pointing toward a heterotrophic lifestyle under symbiotic conditions (Dixon and Kahn, 2004). The general proteome analysis of bacteroids is compared with *in vitro* grown cells in order to identify nodule specific adaptations, over time or when plants were exposed to drought stress (Delmotte et al., 2010; Nomura et al., 2010). ABC-type transporters was present in nodule bacteria for transport of amino acids and inorganic ions along with proteins involved in vitamin synthesis, fatty acid, nucleic acid, cell surface synthesis, and stress-related processes (Sarma and Emerich, 2006). Integrated proteomics and transcriptomics data for *B. japonicum* bacteroids resulted in 2315 proteins involved in carbon and nitrogen metabolism, including a complete set of tricarboxylic acid cycle enzymes, gluconeogenesis and pentose phosphate pathway enzymes, along with other proteins important in symbiosis. Amino acids (Asn, Gln, Pro), organic acids (threonic acid), sugars (Rib, maltose), and polyols (mannitol) were reported to be more abundant in symbiotic roots (Delmotte et al., 2010). In planta studies is very effective to study the interactions between plant and bacteria.

## Gene regulation in symbiotic bacteria

An effective nitrogen-fixing symbiosis establishes as a result of a complex molecular interplay between both partners that involves bacteria-plant signal exchanges and intricate signaling processes (Dixon and Kahn, 2004). Rhizobia start as epiphyte attaches to the root hair, penetrate through the epidermis, divide within the plant-derived infection thread and then invade the cortical cells. On the other hand, in response to the bacterial infection, the plant cortical cells form a new organ, the nodule, where the bacteria differentiate into bacteroids and commence nitrogen fixation. An increased abundance of proteins involved in protein synthesis and degradation was observed during the early stage of nodule development, which may be explained by a restructuring of the proteome to attain a symbiotic lifestyle. Transformation of a free-living bacterium to a nitrogen-fixing endosymbiont results in significant physiological and developmental changes in the rhizobia, including the expression of nif and fix genes, which encode the proteins involved in the nitrogen fixation process in later stages of infection (Karunakaran et al., 2009; Nomura et al., 2010). To withstand changing environmental conditions, the bacteria must possess the ability to undergo specialized physiological adaptations. These response mechanisms are complex and remain largely unexplored. Comparative proteomic display of methylobacterium grown *in vitro* and in planta leads to identification of metabolic proteins and proteins involved in stress response such as the extracellular protease HtrA, SOD, catalases, and the DNA protection protein. In this study, also a key regulator, PhyR, was identified and shown to be essential for phyllosphere colonization (Gourion et al., 2006). These proteins seem to be important for the symbiotic relationship of microbe with plant.

**Figure 1:**
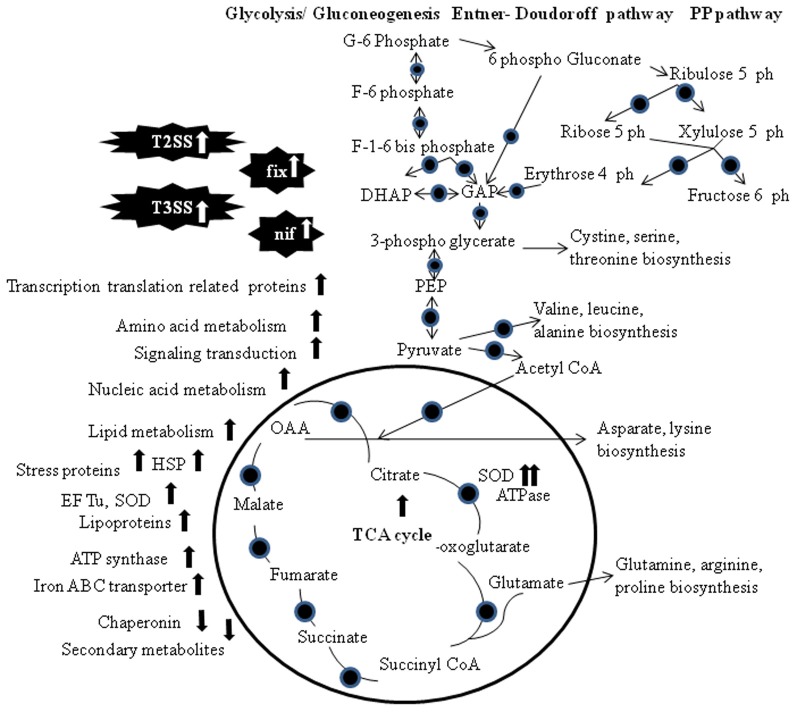
**Visualization of metabolic and proteomics pathways that may supply symbionts with energy under symbiotic conditions.** The TCA cycle, gluconeogenesis/glycolysis, Entner–Doudoroff pathway and the Pentose Phosphate pathway are shown. Circles indicate proteins that have been detected at the transcriptomics and/or proteomics level. Abbreviations: PP, Pentose Phosphate; DHAP, dihydroxyacetone phosphate; GAP, glyceraldehyde-3-phosphate; PEP, phosphoenolpyruvate; OAA, oxaloacetate; G, glucose; F, fructose; EF-Tu, elongation factor thermo unstable; SOD, superoxide dismutase; HSP, heat shock protein; T2SS, type II secretion system; T3SS, type III secretion system.

Proteomic and transcriptomics approach was used to identify genes whose expression is regulated by the NifA-RpoN system in *Rhizobium etli* in symbiosis with *Phaseolus vulgaris* (Salazar et al., 2010; Figure 1). Twenty four proteins associated with NifA and RpoN motifs were identified via differentially proteomic analysis of mutant and wild type *R. etli* strain CFN42. Transcriptomics analysis helps to identify proteins with isoelectric points or molecular weights outside the electrophoretic resolution range of the 2-DE. NifA is a regulator for nitrogen fixation genes in symbiotic diazotrophic bacteria, and controlled by the oxygen-responsive two-component FixL-FixJ/Redox-sensing system RegS-RegR (Dixon and Kahn, 2004). Two further studies support regulatory role of Hfq in symbiosis process in *S. meliloti* (Barra-Bily et al., 2010; Torres-Quesada et al., 2010). Hfq is suspected to contribute to control central metabolic pathways in free-living bacteria and affects survival and nitrogen fixation capacity of the symbionts in the nodules, probably via the regulation of nifA and fixK1/K2 (Torres-Quesada et al., 2010). Barra-Bily et al. (2010) found that oxidative stress proteins and proteins involved in RpoE regulation were down regulated in hfq mutant. High expression of TonB-dependent receptors involved in transport processes of various carbohydrates was reported in *Sphingomonas* (Delmotte et al., 2009).

Hauberg et al. (2010) reported the PilR regulated cascade differentially present in symbiont *Azoarcus* sp. strain BH72 as compared to its mutant. It may be explained by a restructuring of the proteome to attain a symbiotic lifestyle. Koch et al. (2010) discussed host adaptation of *B. japonicum* in nodules of soybean, cowpea or siratro. Transcriptomics and proteomics analyses resulted in the identification of seven unique gene products that were the house keeping gens, along with ABC transporter, substrate-binding protein and a monooxygenase. In planta studies impart PhyR regulation along with the role of T3SS in infection process. The infection process resulted in augmentation of enzymes involved in gluconeogenesis, glycolysis, pentose pathway, amino acid, nucleic acid, lipid metabolism along with stress related proteins.

Protein secretion of a symbiont (*Sinorhizobium meliloti*) and a pathogen (*P. syringae* DC3000) in the presence of root exudates from *A. thaliana* and *Medicago sativa* was studied. SOD is known to be necessary for symbiotic properties in *S. meliloti* and had higher expression in interaction with *M. sativa* (De-la-Pena et al., 2008). Extracellular proteins of the nitrogen-fixing symbiont *Frankia* were analyzed by dissecting nodules of infected cortical cells (Mastronunzio et al., 2009). The secreted proteins within the pool of identified proteins were recognized based on their export signal peptides by *in silico* prediction. Proteins detected in the secretome of three different *Frankia* strains were mainly solute-binding proteins, underlining the importance to acquire nutrients during symbiosis.

## Virulence factors and secretions systems (SS) involved in plant infection

The genes encoding vir and symbiosis determinants are carefully regulated in order to prevent dissipative or premature expression. The expression of these genes is regulated by transcriptional regulators, which are part of regulatory systems such as the quorum sensing system and respond to different extracellular stimuli for instance, nutrients, oxygen levels, presence of plant-derived molecules or population density (Buttner and Bonas, 2010). The concerted action of different regulatory systems can vary substantially, even between closely related strains (Seo et al., 2008). A major task in understanding the regulation of gene expression is the identification of genes that are under the control of distinct regulators. Usually, transcriptomics studies are performed to address this question; however, proteomic analyses are also used, in particular when the aim is the evaluation of the impact on secreted proteins.

The establishment of a symbiotic or pathogenic association is largely dependent on the interaction between the microorganism and the host plant via secreted proteins. Effector proteins secreted by the pathogenic bacteria, either into the extracellular milieu or directly into the host cell cytosol target the host. The proposed biological roles and the mode of action of various effectors in plant pathogenicity have been reviewed (Mudgett, 2005). Combination of SS is involved in process of pathogenesis. In *Xanthomonas* spp six types of protein SS (I–VI) are involved in pathogenesis process (Bonemann et al., 2010). Effector proteins interfere with the signaling cascades of susceptible hosts to counteract, for the plant innate immune responses, which are triggered by PAMPs (Mudgett, 2005). Proteomic studies were frequently applied to analyze the spectrum of proteins secreted by a particular system or to identify the system by which a certain protein is transferred.

**Figure 2:**
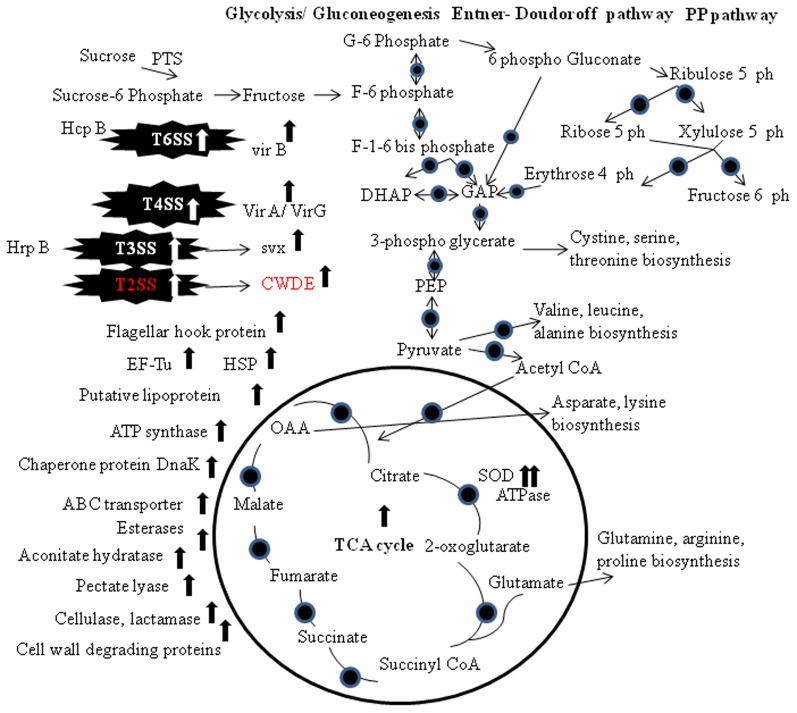
**Biochemical pathways of the pathogen secretion system and proteins expression.** The TCA cycle, gluconeogenesis/glycolysis, Entner–Doudoroff pathway and the Pentose Phosphate pathway are shown. Circles indicate proteins that have been detected at the transcriptomics and/or proteomics level. Abbreviations: PP, Pentose Phosphate; PTS, phosphoenol pyruvate-carbohydrate phosphotransferase system; G, glucose; F, fructose; PEP, phosphoenol pyruvate; TCA, tricarboxylic acid; OAA, oxaloacetate; T6SS, type VI secretion system; T4SS, type IV secretion system; T3SS, type III secretion system; T2SS, type II secretion system; EF-Tu, elongation factor thermo unstable; SOD, superoxide dismutase; HSP, heat shock protein; CWDE, cell wall degrading enzymes.

The co-regulation of the synthesis of enzymes secreted via the T2SS and T3SS has been reported frequently (Figures 1, 2). One reasonable explanation for this co-regulation is based on the observation that substrates that are secreted via the T2SS are not only associated with bacterial vir but can also induce plant defense responses. The concurrent induction of the T2SS and the T3SS probably allows the pathogen to counteract basal plant defense responses that are elicited by proteins released via the T2SS with the effector proteins transported by the T3SS (Jha et al., 2007). T3SS are used to inject different effector proteins, formerly known as avirulence proteins, directly into the host cell via an extracellular pilus that acts as channel (McCann and Guttman, 2008). T3SS is required for pathogenicity in host plants and for eliciting a rapid hypersensitive resistance response in non-host plants, and the genes encoding T3SS were named HR and pathogenicity (hrp) genes (Jacobs et al., 2012). hrp genes were identified in most Gram-negative plant pathogenic bacteria with the exception of *A. tumefaciens* and *Xylella fastidiosa* (Buttner and Bonas, 2010). Homologues of the hrp genes were also found in different rhizobia (Jones and Dangl, 2006). To identify the SS that is responsible for the transport of distinct vir factors, the secretome of a SS mutant strain and the wild-type strain is compared (Kazemi-Pour et al., 2004; Watt et al., 2009). Joshi et al. (2010) discussed twin arginine transport (Tat) dependent translocation of vir factor in *S. scabies* under T3SSs. Tat-secreted vir proteins includes lipoproteins, ABC transporters, phospholipases/phosphoesterases, β-lactamase, and proteins involved in Fe homeostasis and Tat dep vir factors (Table 1). T3SS-secreted proteins responsible for vir process usually silenced in plant-pathogenic bacteria and were only induced during the plant infection process (Buttner and Bonas, 2002). Another vir factor Svx (secreted vir factor from *Xanthomonas*), in a secrotomic study of *P. atrosepticum*, was analyzed (Corbett et al., 2005).

Addition of galacturonate, pectin or plant extract to the medium resulted in the induction of *vir* factors in Enterobacteriaceae (Kazemi-Pour et al., 2004; Mattinen et al., 2007). In *Xanthomonas* secretome studies a defined medium with fructose, sucrose and casamino acids was found to induce T3SS gene (Wengelnik et al., 1996; Yamazaki et al., 2008). To study the secretome of *A. tumefaciens*, acetosyringone was added to induce VirB1 (Wu et al., 2008). *P. atrosepticum* in the presence of potato tuber extract, induce vir factors such as pectic enzymes, proteinase, and the avirulence protein homolog Svx (Mattinen et al., 2007). Likewise, culturing *X. campestris* pv. campestris in the presence of *B. oleracea* led to the induction of a nuclease, a ribonuclease, peptidases, proteases and cell-wall-degrading enzymes (Watt et al., 2009). T3SS was induced by addition of flavonoid (genistein) to the medium, resulted in secretion of the nodule outer proteins (Nops), which is involved in the host-range determination (Jones and Dangl, 2006).

Protein secretion into the extracellular space or other space was mediated by outer membrane vesicles (OMVs) in some bacterial strains. OMVs are an ideal structure to transport hydrophobic compounds like membrane proteins into host cells or quorum signals to neighboring cells (Wai et al., 2003; Mashburn and Whiteley, 2005). OMVs of gram-negative bacteria contain outer membrane proteins, periplasmic proteins, lipopolysaccharides, phospholipids, DNA, toxins, and other factors associated with vir (Kadurugamuwa and Beveridge, 1997; Mashburn and Whiteley, 2005). They are known to be constantly liberated from the outer membrane and are thus a likely source of outer membrane proteins in the culture supernatant (Kadurugamuwa and Beveridge, 1997). In the presence of vir factor inducing medium, two major protein groups were detected: outer membrane proteins and vir -associated proteins. The latter group included proteins of the T3SS, effector proteins, two cell wall-degrading enzymes, a cellulase, and a xylosidase.

Several periplasmic proteins were detected in the OMVs, which may be entrapped in the vesicle lumen during their release from the outer membrane. One of the identified vir proteins is HrpF, the putative translocon of the T3SS that is proposed to be inserted into the host membrane and serves as attachment site for the T3SS conduit. It is currently assumed that this protein is transported via the T3SS and then pushed into the host membrane (Buttner and Bonas, 2002). However, since OMVs of mammalian pathogens have been reported to deliver vir -factors into host membranes by fusion (Wai et al., 2003), it can be suspected that HrpF is inserted into the plant membrane by fusion of OMVs carrying HrpF.

Proteomic studies confirmed the transcriptional activation of vir factor (Hrp) via T2SS in *Burkholderia glumae* and *X. axonopodis* pv. citri (Kang et al., 2008; Yamazaki et al., 2008). Vir factor Svx was secreted via the T2SS in *P. atrosepticum* (Corbett et al., 2005). Protein secretion in the Gram-positive *S. scabies* via the twin arginine transport (Tat) pathway was analyzed. In Gram-negative bacteria the Tat system is part of the T2SS (Johnson et al., 2006). T2SS are present in many gram-negative proteobacteria, and toxins and extracellular enzymes such as proteases, lipases, and cell-wall-degrading enzymes were secreted via the T2SS (Jacobs et al., 2012). Components of the T2SS were identified as vir factors in the plant pathogenic bacteria *D. dadantii, Pectobacterium carotovorum, Xanthomonas* spp., and *Ralstonia solanacearum* (Buttner and Bonas, 2010). Comparison of the extracellular proteome of a tatC mutant and the wild type in combination with an *in silico* search for proteins harboring a secretion signal identified 73 predicted secretory proteins whose expression was reduced in the mutant. The tatC mutant strain was almost completely avirulent, indicating that the activity of this transport system is mandatory for vir. Hemolysin- coregulated proteins (Hcp) vir factor is structural component of a type T6SSs, components of the T4 bacteriophage tail tube and is required to puncture the host membrane in the context of phage infection (Bonemann et al., 2010). Hcp is transported via the T6SS in *A. tumefaciens* (Wu et al., 2008). Comparative proteome analysis with and without acetosyringone confirmed the induction of Vir proteins comprising the type IV secretion system for the delivery of bacterial T-DNA into the plant host cell. More induced proteins were identified, one of these is a molecular chaperone and functions as an assembly factor for the type IV secretion system (Lai et al., 2006; McCann and Guttman, 2008). The proteins that enable successful infection of plants are termed vir factors. These include cell surface proteins such as adhesins, polysaccharides, lipopolysaccharides, and degradative enzymes such as cellulases, pectate lyases, and proteases that enable the degradation of the plant cell wall under the action of T2SS (Jacobs et al., 2012; Figure 2).

## Proteomics analysis of the plants affected by bacteria

Inspite of lot of work on plant-microbe interactions, there are still lot of gaps in different proteomic studies of plants in response to bacterial infection. Here there is brief preview of some of research articles regarding plant responses to bacterial attack, which can be helpful to understand the plant microbe interactions (Table 2). Miao et al. (2008) reported that caffeoyl CoA 3-O-methyltransferase gene was down regulated in susceptible cultivars of tomato in response to inoculation with bacteria. Defense-related antioxidants such as pathogenesis-related (PR) -9 and metabolic enzymes were reported in *A. thaliana* in response to *P. syringae* (Jones et al., 2004, 2006a). Both of these groups of antioxidant enzymes were considered to have probable significant roles in the regulation of redox conditions within infected tissue. Many new techniques such as hexapeptide ligand libraries (CPLL such as proteominer) had been used to decrease the high abundant proteins for enrichment of low abundant protein. Frohlich et al. (2012) applied the CPLL in *A. thaliana* leaf proteins after infection with virulent *P. syringae*. 2-DE showed a decrease in high-abundance proteins and an enrichment of less abundant proteins in leaf samples. Mass spectrometric analyses of leaf extracts led to the identification of 312 bacterial proteins in infected *Arabidopsis* leaves.

**Table 2:** **Proteomics analysis of plants in response to pathogenic/symbiotic bacteria**.

**Studied organism**	**Pathogen**	**Proteomic approach**	**No. of IP**^**1**^	**Identified proteins**	**References**
*A. thaliana*	*P. syringae*	2-DE LC MS/MS	52	Abundant proteins (defense-related, Transcription, elongation factor, peroxiredoxin, arginase, lipase/acylhydrolase, PS11, sedoheptulose bisphosphatase, Protein folding and turnover, cytochrome, Cellular transport and ion homeostasis, decreased proteins, metabolism, and proteasome.	Jones et al., 2006a
*A. thaliana*	*P. syringae*	iTRAQ	5	Phosphoproteome changes 4 proteins (dehydrin, co-chaperone, heat shock protein, plastid-associated protein) and ribulose-1, 5-bisphosphate carboxylase/oxygenase large subunit (RuBisCO LSU).	Jones et al., 2006b
*A. thaliana*	*P. syringae*	2-DE; LC MS/MS	2	Glutathione S-transferase (GST), peroxiredoxin.	Jones et al., 2004
*O. sativa* transgenic and/or inoculated with *Xanthomonas oryzae* pv. *oryzae*	*X. oryzae pv. oryzae*	2-DE MS/MS Protein sequencer	10	Accumulation of pathogenesis-related protein (PR) -5, superoxide dismutase, peroxiredoxin, glycine cleavage H protein, glyceraldehydes 3-phosphate dehydrogenase, triose phosphate isomerase, oxygen evolving complex is downregulated.	Mahmood et al., 2006
*O. sativa*	*X. oryzae pv. oryzae*	2-DE, MS/MS	20	Defense-related plasma membrane proteins with increased abundance are ATPase, phosphatase, hypersensitive response, prohibitin, zinc finger and C_2_ domain protein, universal stress protein, heat shock protein, ascorbate peroxidase (APX), alcohol dehydrogenase, and quinone reductase.	Chen et al., 2007
*O. sativa*	*X. campestris* pv. Oryzicola	2-DE, MALDI-TOF MS	32	PR-1, PR-10, receptor-type protein kinase, ascorbate peroxidase, adenine triphosphate (ATP) synthase, RuBisCO LSU, ribonuclease, phospholipase, and GTP binding protein.	Li et al., 2012
*L. hirsutum* (leaflets)	*Cl. michiganensis* ssp. michiganensis	2-DE, ESI-MS/MS	47	Regulatory proteins accumulated: defense and stress related (PR-3, GST, APX, superoxide dismutase), regulatory proteins, protein synthesis and processing (chaperonin, elongation factor-thermo unstable), carbon metabolism: RuBisCO (LSU, small subunit, activase, epimerase, triose phosphate isomerase), metabolism [glycine cleavage system, oxygen evolving enhancer (OEE)], and ATP production [nucleotide diphosphate kinase (NDK), ATP synthase].	Coaker et al., 2004
*M. truncatula, root*	*S. meliloti*	2-DE, PMF	99	Proteins increased in abundance: defense and stress related (PR-9, -10, APX, superoxide dismutase), S-adenosyl methionine synthase, GST, elongation factor, NDK, protein disulfide isomerase (PDI), OEE, protein synthesis and degradation, isoflavone reductase, hormone dependent proteins, and metabolic proteins (ATP synthase, fructose bisphosphate aldolase).	Mathesius et al., 2003
*S. lycopersicum*	*P. solanacearum*	Protein sequencer	15	Proteins highly accumulated: protein destination and storage (60 kDa chaperonin, PDI, heat shock protein), protein synthesis, metabolism (RuBisCO activase, plastocyanin, glycine dehydrogenase, OEE), and defense (calgranulin, AMA).	Afroz et al., 2009
Rice (var. Co43)	*P. fluorescens* KH- 1 (PGPR)	2-DE, MS, LC MS/MS	23	Highly abundant proteins of energy metabolism, photosynthesis, protein degradation and antioxidation: GST, NDK, chaperone, thioredoxin, RuBisCO LSU, and proteosome	Kandasamy et al., 2009
*Malus Domestica* cv. Holsteiner Cox	*P. fluorescens*	SDS-PAGE, MS/MS	5	PR-2, -3, -4b, -5, -10, ribonuclease-like, endochitinase class III.	Kurkcuoglu et al., 2004
*S. lycopersicum*	*P. solanacearum*	Protein sequencer	15	Proteins with increased accumulation in response to JA and SA: defense (S-adenosyl methionine synthase, arginase, peroxiredoxin, threonine diaminase, polyphenol oxidase, leucine aminopeptidase), protein synthesis (PDI), protein destination and storage (60 kDa chaperonin), energy (glycine cleavage, ATP synthase), and RuBisCO LSU downregulated.	Afroz et al., 2009
*G. max*	*B. japonicum* (symbiotic root nodules)	GC MS, LC-QTOF-MS	166	Genes highly expressed: fatty acid, amino acid, carboxylic acid metabolism, disaccharides, isoflavonoids, and glucosinolate.	Brechenmacher et al., 2010

1 IP: Number of identified protein.

Abbreviations: ATP, adenine triphosphate; GST, glutathione S-transferase; RuBisCO, ribulose-1, 5-bisphosphate carboxylase/oxygenase; LSU, large subunit; APX, ascorbate peroxidase; PR, pathogenesis-related protein; OEE, oxygen evolving enhancer; PDI, protein disulfide isomerase; NDK, nucleotide diphosphate kinase; 2-DE, two-dimensional polyacrylamide gel electrophoresis; MS, Mass spectrometry; MS/MS, Tandom Mass spectrometry; LC, liquid chromatography; GC, gas chromatography; MALDI-TOF, Matrix-assisted laser desorption/ionization time of flight.

Accumulation of free linolenic and benzoic acid or reduction in lauric acid was found to be important indicator of an active plant defense response in *G. max*. γ-aminobutyric acid, proline, and glutamine reduction resulted in *G. max* susceptibility after *Bradyrhizobium japonicum* inoculation (Brechenmacher et al., 2010). Transcriptomic and proteomic approaches identified numerous genes and proteins involved in carbon and nitrogen metabolism, plant defense responses, nutrient exchange, and signal transduction that are significantly regulated in *G. max* colonized by *B. japonicum* (Brechenmacher et al., 2010). Li et al. (2012) discussed importance of PR-1 and PR-10 in rice defense against *Xanthomonas campestris*. Components of PSII, mitochondrial permeability transition and cytoplasmic antioxidant enzymes were modified during R-gene-mediated HR. PR-9 and PR-5 were induced in response to *X. oryzae* pv. Oryzae in rice (Mahmood et al., 2006). Analyses clearly revealed that four defense-related proteins (PR-5, Probenazole-inducible protein, SOD and peroxiredoxin) were induced for both compatible and incompatible *X. oryzae* pv. Oryzae races, where PR-5 and probenazole-inducible protein were more rapid and showed higher induction in incompatible interactions and in the presence of JA. Furthermore, the sense PR-5 transgenic rice plants were more resistant than the susceptible vector control against *X. oryzae* pv. Oryzae (Mahmood et al., 2009).

Chen et al. (2007) analyzed proteins from rice plasma membrane to study the early defense responses involved in *Xa21* mediated resistance. *Xa21* is a rice receptor kinase, which is predicted to perceive the *X. oryzae* pv. oryzae signal at the cell surface, leading to the “gene-for-gene” resistance response (Song et al., 1995). Twenty proteins that were differentially expressed had potential functions in rice defense. Proteins expressed in partially resistant lines and a susceptible tomato species that are regulated in response to *C. michiganensis* ssp. michiganensis were identified 72 and 144 h post inoculation. Using 2-DE and MS, 26 differentially regulated tomato proteins were identified; 12 of which were directly related to defense such as PR-3, PR-9, and stress responses (Coaker et al., 2004). PR proteins seem to be good candidates to develop the incompatible interactions with bacteria along with SAR in plants. Proteomic analysis was also used to detect the responses of the model legume *M. truncatula* to the pathogenic bacterium *P. aeruginosa* (Mathesius et al., 2003). There is accumulation of 154 proteins, among which 21 are related to defense and stress responses. Molecular chaperones, protein related to defense, destination and storage were differentially expressed in resistant tomato cvs (Afroz et al., 2009). Apical membrane antigen was found to be the novel protein expressed in susceptible cultivar in SA cascade (Afroz et al., 2010). Kandasamy et al. (2009) reported differential proteins in response to *P. fluorescens* in rice leaf sheath (Table 2). Along with protein degradation and antioxidation, photosynthetic proteins thioredoxin was upregulated. Defense proteins can be the other target protein for the disease resistance against bacterial pathogens. Tomato seed treatment with rhizobacteria *P. fluorescens* exhibited growth promotion along with protection from infection by *Fusarium oxysporum* (Ramamoorthy et al., 2002). PR-2, PR-3, and PR-5 were found to be induced in *P. fluorescens* treated plants challenged with *F. oxysporum*. Similarly, the expression of glutathione S-transferase (GST) is known to be involved in tagging toxic endogenous substrates with glutathione conjugation to transport toxic substrates into the vacuole through a glutathione pump (Ishikawa, 1992). GST is reported to be induced in response to *P. syringae* in *Arabidopsis*, and has an important role in plant defense from oxidative damages caused by various biotic or abiotic stresses such as heavy metal, wounding, ethylene, ozone, and pathogen attack (Marrs, 1996; Jones et al., 2004, 2006a,b).

Chloroplasts may be key players in plant defense, as the loss of integrity of PSII may lead to the HR-associated oxidative burst, thereby restricting pathogen growth. Proteins related to photosynthesis, defense, protein destination and storage had been down regulated in response to *P. syringae* infection in soybean (Zou et al., 2005). Proteins differentially expressed in response to *P. savastanoi* pv. savastanoi on *O. europaea* stems were related with photosynthesis and metabolism (Campos et al., 2009). Non-pathogenic bacteria (*P. putida*) had been reported to promote systemic resistance in different host plants as well. Stimulation of antifungal material (phytoalexin) and proteins in lipoxygenase pathway in tomato were found after infection with *P. putida* (Akram et al., 2008).

Holzmeister et al. (2011) used 2-D fluorescence difference gel electrophoresis technology to demonstrate the role of niric oxide in plant defense signaling. After infection of wild type and *Arabidopsis* mutant strain, it was found that in the infection by avirulent *P. syringae* there is accumulation of defense, redox as well as stress proteins, while in virulent infection only defense proteins were expressed. This imparts importance of stress proteins in plant defense signaling. Induction of defense enzymes involved in phenyl propanoid pathway and accumulation of phenolics and PR-proteins might have contributed to restriction of invasion of *F. oxysporum* f. sp. lycopersici in tomato roots. Induction of defense proteins and chemicals in tomato by *P. fluorescens* isolate against *F. oxysporum* was studied. Defense-related proteins such as PR-9, PR-2, PR-5, and 46 kDa chitinase increased in bacterized tomato root tissues (Ramamoorthy et al., 2002). Proteins related to photosynthesis, defense, destination, and storage are decreased as a result of bacterial infection followed by the induction of PR proteins in JA or SA pathway. Defense-related proteins, PR proteins and PSII destruction and repair system can be the candidates for improved resistance against bacterial infection in plants.

## Conclusions

This review summarizes the use of proteomic approaches to understand the molecular and cellular processes that govern host responses such as PTI, PRR, and ETI. Detailed global comparison of response pathways using proteomics has allowed the identification of novel proteins whose biological role warrants in-depth biochemical and cellular elucidation. Finally understanding of biotic stress responses may identify promising novel targets for the development of cultivars with improved disease resistance. Various types of bacteria can trigger rapid responses in plant cell cultures and defense responses in intact plant tissues (Jones and Dangl, 2006). Flagellin and lipopolysaccharides have been identified as common bacterial determinants or PAMPs that act as elicitors of defense responses in plant cells (Felix et al., 1999). R genes that confer resistance to various phytopathogens are proposed to be part of a positive feedback loop to amplify the response triggered by PAMP perception. ETI is often accompanied by local cell death known as the HR illustrating the dynamic evolution between plants and pathogens (Martin et al., 2003; Jones and Dangl, 2006).

To fully elucidate microbial metabolism and its responses to environmental factors, it will be necessary to go beyond the information obtained from proteomic studies alone. There is need of integration of data resulting from the functional characterization and quantification of molecules representing from genes, transcripts and proteins to metabolites (Delmotte et al., 2010). First steps in this direction were made by setting up extensive proteomic reference databases and by compiling proteomic and transcriptomics data sets (Bosch et al., 2008; Koch et al., 2010). To profit from such analyses as best as possible, databases should be set up that combine and integrate the results obtained in different studies. With complete genome sequences of several host and pathogen partners now available, there are literally hundreds of candidate genes and proteins with potential applications in crop protection. Knowledge generated from research such as described in this special issue will open new avenues for the engineering of durable resistance to bacterial pathogens in plants. Identified proteins involved in plant tolerance to stresses could be used for development of disease resistant cultivars as well as these proteins can be used as markers for the identification of type of infection.

### Conflict of interest statement

The authors declare that the research was conducted in the absence of any commercial or financial relationships that could be construed as a potential conflict of interest.

## Abbreviations

PAMP: pathogen associated molecular pattern
PRRs: pattern recognition receptors
PAB: plant-associated bacteria
ETI: effector triggered immunity
R: resistance proteins
HR: hypersensitive response
EF: Elongation factor
T2SSs, T3SSs, T4SSs, T6SSs: type 2,3,4,6 secretion systems
LRR: leucine-rich repeat
EFR: elongation factor receptor
FLS2: flagellin sensing 2
vir: virulence
SA: salicylic acid
JA: jasmonic acid
MAP: mitogen-activated protein
2-DE: two-dimensional polyacrylamide gel electrophoresis
MS: mass spectrometry
MALDI-TOF: matrix-assisted laser desorption/ionization time of flight
LC: liquid chromatography
MS/MS: tandom mass spectrometry
SOD: superoxide dismutase
ROS: reactive oxygen species
hrp: hypersensitive reaction and pathogenicity gene
RuBisCO: ribulose-1, 5-bisphosphate carboxylase/oxygenase
PR: pathogenesis-related
GST: glutathione S-transferase
Hcp: hemolysin- coregulated proteins
OMVs: outer membrane vesicles
CPLL: hexapeptide ligand libraries.
